# POST: photonic swin transformer for automated and efficient prediction of PCSEL

**DOI:** 10.1515/nanoph-2025-0317

**Published:** 2025-09-30

**Authors:** Qi Xin, Hai Huang, Chenyu Li, Kewei Shi, Zhaoyu Zhang

**Affiliations:** School of Science and Engineering, The Chinese University of Hong Kong, Shenzhen, Guangdong 518172, China; Guangdong Key Laboratory of Optoelectronic Materials and Chips and Shenzhen Key Lab of Semiconductor Lasers, School of Science and Engineering, The Chinese University of Hong Kong, Shenzhen, Guangdong 518172, China; The University of Hong Kong, Hong Kong, China

**Keywords:** deep learning, vision transformer, photonic crystal, coupled-wave theory

## Abstract

This work designs a model named POST based on the vision transformer (ViT) approach. Across single, double, and even triple lattices, as well as various non-circular complex hole structures, POST enables prediction of multiple optical properties of photonic crystal layers in photonic crystal surface emitting lasers (PCSELs) with high speed and accuracy, without requiring manual intervention, which serves as a comprehensive surrogate for the optical field simulation. In the predictions of quality factor (*Q*) and surface-emitting efficiency (SE) for PCSEL, the R-squared values reach 0.909 and 0.779, respectively. Additionally, it achieves nearly 5,000 predictions per second, significantly lowering simulation costs. The precision and speed of POST predictions lay a solid foundation for future ultra-complex model parameter tuning involving dozens of parameters. It can also swiftly meet designers’ ad-hoc requirements for evaluating photonic crystal properties. The database used for training the POST model is derived from predictions of different photonic crystal structures using the coupled-wave theory (CWT) model. This dataset will be made publicly available to foster interdisciplinary research advancements in materials science and computer science.

## 1 Introduction

Photonic crystal surface emitting lasers (PCSELs) are a novel type of semiconductor lasers that achieve high power, high beam quality, and low divergence by applying a two-dimensional photonic crystal layer as optical resonant cavity [1], [2]. The periodic modulation of the refractive index enables in-plane distributed feedback and vertical radiation, making PCSELs advantageous over traditional VCSELs in terms of scalability and output coherence [3], [4], [5]. Conventional modeling techniques for EELs and VCSELs – such as Fabry–Pérot cavity analysis or 1D transfer matrix methods – are efficient for vertically layered structures but fail to capture the lateral periodicity and complex mode coupling in PCSELs. Numerical solvers like FDTD (Finite-difference time-domain) can handle these effects, but their high computational cost (hours per simulation) limits scalability [4], [6], [7]. Coupled-wave theory (CWT) offers a more efficient alternative. By expanding electromagnetic fields into spatial harmonics, CWT accurately models in-plane diffraction and vertical radiation in photonic crystals [8], [9], [10]. It provides a good balance between physical accuracy and computational speed, making it especially suitable for large-scale PCSEL design [1].

While CWT is significantly faster than full-wave solvers, it still requires several minutes per simulation for complex PCSEL unit cells – especially those with multi-lattice or irregular hole geometries: Conducting 100,000 simulations will take nearly a year. Moreover, irregular geometries lead to the explosion of the number of design variables – potentially dozens or even over one hundred, which increases the volume of the simulation space exponentially. Optimization process on such space always requires millions or even billions of simulations.

To overcome these challenges, an idea is to establish a database where a machine learning architecture can learn the physical principles of PCSEL’s photonic crystal layers and makes efficient predictions, for example, within 0.001 s per sample. Then over 80 million predictions could be performed daily to satisfy the requirements of high-dimensional optimization.

Vision Transformer (ViT) architecture is the go-to solution for this machine learning problem. Inspired by the success of Transformers in natural language processing (NLP) [11], [12], [13], [14], [15], researchers began exploring their application to computer vision. ViT [16] proposed a purely transformer-based architecture for image classification, challenging the dominance of convolutional neural networks (CNNs). ViT divides an image into fixed-size patches (e.g., 16 × 16), treats each patch as a token, and processes the resulting sequence using standard transformer mechanisms, mirroring how sequences of words are handled in NLP. ViT has demonstrated strong performance on image classification [16], [17], object detection [18], [19], [20], [21], and semantic segmentation [22], [23], [24], [25], and has been extensively improved through works such as DeiT [26] and ConViT [27] to enhance its training efficiency and accuracy. ViT offers advantages like lower computational cost and compatibility with transformer-based optimization frameworks.

The swin transformer (SwinT) [28] is one of the ViT models. It computes self-attention within non-overlapping local windows, reducing complexity from quadratic to linear. The introduction of shifted windowing facilitates cross-window information flow and enhances local context modeling. These architectural innovations make SwinT a versatile backbone for a wide range of vision tasks and are key to its success in our proposed POST model.

Despite ViT’s advantages, there has been limited application of ViT architectures to physical modeling tasks, particularly in photonics. Accurate modeling of the optical properties of complex PCSEL photonic crystal layers remains largely unexplored. This presents a significant research gap and a promising direction for applying ViT-based methodologies to advanced physical modeling and predictive tasks in photonics [29], [30], [31]. The application of ViT in photonic crystal design faces challenges: ViT model has high data requirements, difficulties in visual reasoning and training stability issues, while design of photonic crystal requires simultaneously analyzing both the global and local physical properties effectively.

**Figure 1:**
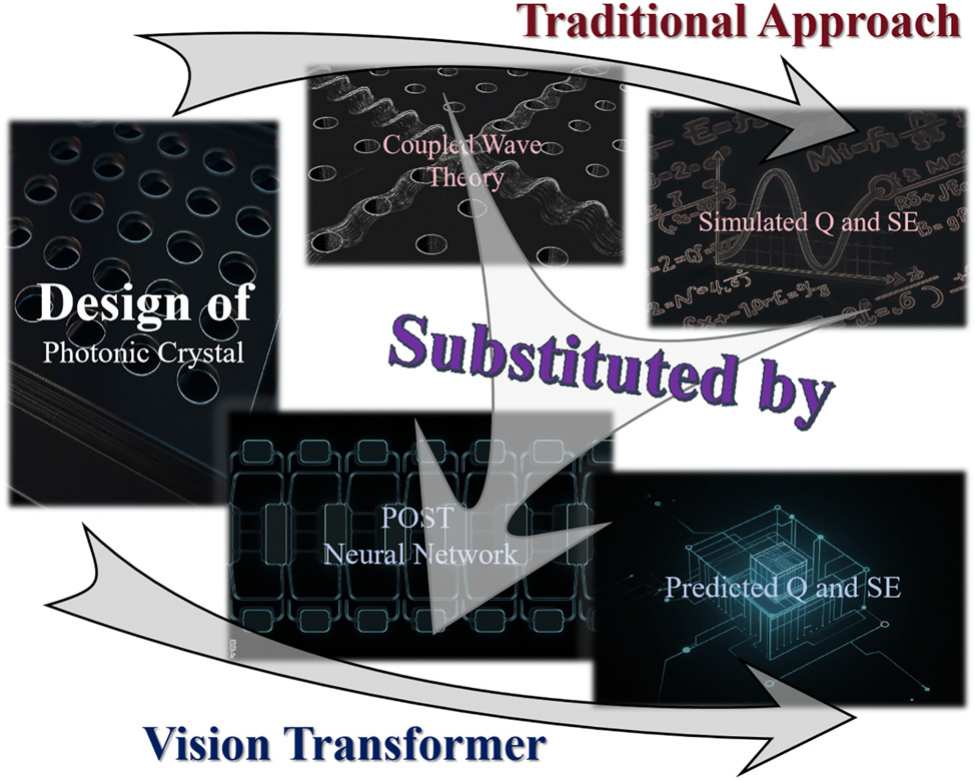
Schematic overview. This work replaces PCSEL’s conventional simulation model with POST neural network prediction model, achieving a qualitative leap in the speed of design characterization.

This work addresses these challenges by employing the CWT model and the POST model (The overview of this work is shown in Figure 1). The POST model handles single-, double-, and triple-lattice configurations as well as arbitrarily shaped holes with smooth curved contours, supporting irregular geometries beyond circles or triangles. POST predicts multiple optical properties efficiently with high accuracy, achieving a simulation speed of about 5,000 samples per second and a training time under 1,000 s per epoch. POST is based on SwinT [28], a state-of-the-art ViT architecture. The mean squared error of predictions is reduced by over 50 % compared to previous works, and it surpasses the prediction accuracy of existing methods with less than 20 % of the original dataset [32].

## 2 Results

### 2.1 Dataset generation

#### 2.1.1 Raw data acquisition via CWT

The epitaxial structure listed in Table 1 serves as a representative baseline for PCSEL design. While the epitaxial configuration influences parameters such as the optical Green’s function and the confinement factor of the photonic crystal (Γ_PhC_) within the CWT framework, its impact on the overall device behavior is secondary to the photonic crystal design. Therefore, the proposed methodology retains broad applicability and can be readily extended to alternative epitaxial stacks without significant modification.

**Table 1:** Epitaxial structure of the PCSEL in this work.

Layer	Material	Thickness (μm)	Refractive index
Photonic crystal	p-GaAs/Air	0.35	3.4826/1
Waveguide	p-GaAs	0.08	3.4826
Electron blocking layers	p-AlGaAs	0.025	3.2806
Active region	InGaAs/AlGaAs	0.116	3.3944
n-cladding	n-AlGaAs	2.11	3.2441
n-substrate	n-GaAs	–	3.4826

To evaluate the optical performance of PCSELs, we adopt the CWT to model the interaction of fundamental waves within the photonic crystal lattice. By considering four primary wave components propagating along orthogonal directions, a set of coupled partial differential equations is established to capture both diffraction feedback and radiation loss mechanisms [33].

Solving this model under appropriate boundary conditions for a finite-size square-lattice photonic crystal allows us to compute the spatial distribution of optical fields, from which key performance metrics can be derived. Among these, two indicators are particularly crucial. Surface-emitting efficiency (SE) defined as the ratio between the surface-emitting optical power and the total stimulated emission power:

(1) $$\mathrm{SE}=\frac{P_{\text{surface}}}{P_{\text{stim}}}=\frac{\alpha_{\text{surface}}}{\alpha_{\text{total}}},$$

where *α*_surface_ and *α*_total_ are the surface radiation loss and total radiation loss of the lasing mode, respectively. This ratio reflects how effectively the laser extracts optical energy through vertical radiation and serves as a direct metric for surface output optimization.

Quality factor (*Q*) quantifies the ratio of stored optical energy to energy lost per oscillation cycle, expressed as:

(2) $$Q=\frac{2\pi /a}{\alpha_{\text{total}}},$$

where *a* is the lattice constant. A higher *Q* indicates better optical confinement and lower lasing threshold.

To ensure consistency across all simulated designs, the Bragg wavelength was fixed at 980 nm by adjusting the photonic crystal lattice constant accordingly, resulting in negligible wavelength variation across the dataset. Similarly, because the structure of the epitaxial layer (Table 1) remains unchanged for all samples, the energy confinement factor does not vary significantly. Furthermore, the analysis specifically targets the fundamental Γ^(2)^-point band-edge mode of the photonic band structure: Variations in other bands or off-Γ^(2)^ modes are beyond the scope of this work. Therefore, focusing on *Q* and SE as prediction targets was a deliberate first step, since these quantities are well defined within the CWT framework and directly reflect device efficiency and feedback strength in the design stage.

Other important figures of merit in PCSEL design, such as device size and the accuracy of the numerical solution to differential equations, were intentionally fixed in this study to avoid introducing additional degrees of freedom that would obscure the model’s evaluation. Nevertheless, POST is inherently data-driven and architecture-agnostic. Provided that the corresponding training data are available, POST can be readily extended to predict emission wavelength shifts, confinement factors under different epitaxial stacks, device size or even full band diagrams. This extensibility ensures that the proposed framework is not limited to *Q* and SE, but can evolve into a comprehensive predictive tool for a broader range of PCSEL performance metrics in future work.

#### 2.1.2 Data preprocessing

**Figure 2:**
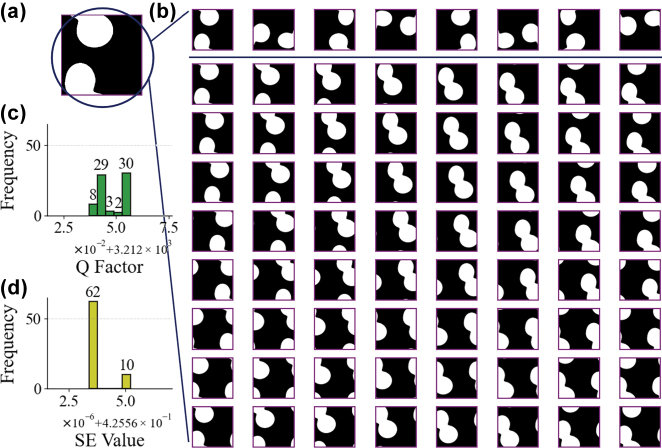
Flip-rotate-translate pattern effects simulation. (a) A randomly generated unit cell pattern of the photonic crystal. (b) The first row shows eight variants of the lattice structure in subfigure after flipping and rotation. Rows two to nine show sixty-four additional patterns generated by horizontal or vertical translation of the original structure. (c–d) Simulation results of coupled-wave theory model for the photonic crystal lattice structure shown in b after flipping, rotation, and translation. The upper and lower histograms show the distributions of simulated *Q* and SE, respectively.

The original dataset used in this work contains 25,000 samples, which we divided into training and test sets at a 4:1 ratio. Since the actual device consists of tens of thousands of lattices arranged in a periodic square matrix, flipping or rotating a single lattice’s design pattern theoretically does not affect the final PCSEL properties. Furthermore, we observed that even translating patterns, which alter the edge structures of the periodic square matrix, has negligible impact on overall PCSEL performance. The accompanying Figure 2 demonstrates this phenomenon using a randomly generated device pattern subjected to flipping, rotation, and translation operations followed by CWT simulations.

The histograms in Figure 2 show highly consistent simulation results in all transformations, with *Q* variations significantly below 1 and SE variations well under 0.1 %. These results not only confirm the pattern-invariant nature of photonic crystal properties but also validate the reliability of the numerical solution component in our CWT model, particularly its convergence characteristics in the non-analytical portion of the calculations.

Physically, the invariance of *Q* and SE under translation arises because a rigid lateral shift of the pattern only introduces a phase change in the Fourier components of the permittivity distribution, without affecting their magnitudes. In the CWT formulation, the key coupling coefficients (*κ*_*i*,*j*_) that determine feedback and radiation are proportional to the magnitude of these Fourier terms and the vertical field profile. Thus, a mere translation, which shifts the phase of each Fourier coefficient *ξ*_*i*,*j*_, leaves the magnitude of *κ*_*i*,*j*_ unchanged. Consequently, the cavity’s mode profile and thus edge losses remain virtually the same under translation, explaining why the *Q* and SE are insensitive to such translations.

Therefore, the dataset size can be expanded using the methods mentioned above to improve prediction accuracy. The related research is discussed in Section 2.3.

**Figure 3:**
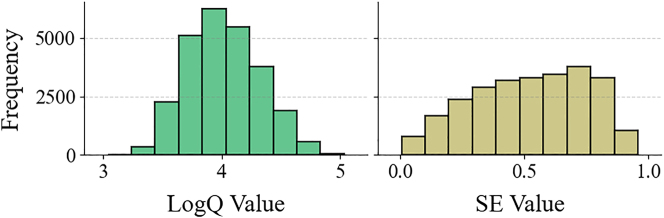
Distributions of data. The histograms show the distributions of log *Q* and SE for all samples in the original dataset.

The choice of prediction targets is also investigated. SE naturally ranges between 0 and 1 with relatively uniform distribution (Figure 3). Results show that raw SE values achieve the highest prediction accuracy without preprocessing (Table 2). In contrast, the Q factor can vary dramatically from hundreds to hundreds of thousands. Taking its logarithm yields a more uniform distribution (Figure 3) and maximizes prediction accuracy (Table 2). Balanced sample distributions across all value ranges enable the neural network to better distinguish between different photonic crystal designs.

**Table 2:** Accuracy versus optimization targets.

Target	*R* ^2^	Target	*R* ^2^
SE	0.779	*Q*	0.636
Std SE	0.775	Std *Q*	0.818
		log *Q*	0.909
		Stdlog *Q*	0.865

This table presents POST’s *R* ^2^ accuracy under different optimization targets. StdQ/StdSE: linear rescaling of all *Q*/SE values to [0, 1] range; Stdlog *Q*: logarithmic transformation of *Q* values followed by [0, 1] rescaling.

### 2.2 POST backbone architecture

#### 2.2.1 Encoding module based on Swin Transformer Block

To enhance the extraction of feature representations from the single-channel input image *I*, we used a Swin Transformer Block-based encoder. The resulting multi-dimensional representation $Z_{4}$ is subsequently passed through an output layer to produce the final prediction results. The overall formulation of the encoding process is expressed as:

(3) $$SwinTransformer\left(I\right)=Z_{4},\;I\in R^{H\times W\times 1}.$$

**Figure 4:**
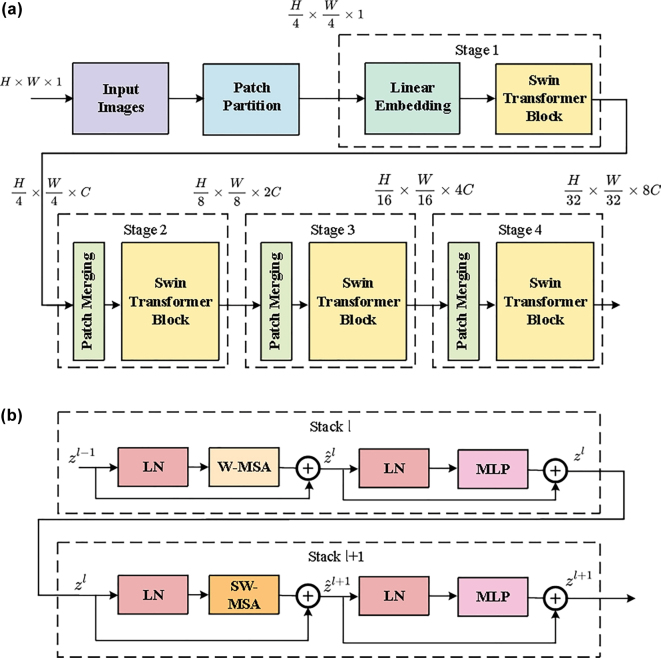
POST network structure. Graph (a) shows the architecture of the SwinT encoder. The input single-channel image is partitioned into patches, linearly embedded, then processed through four hierarchical stages to produce the final multi-dimensional representation $Z_{4}$ . Graph (b) displays two consecutive swin transformer stacks. W-MSA and SW-MSA refer to multi-head self-attention modules with regular and shifted window configurations.

In accordance with the requirements of our task, we adopted the architecture illustrated in Figure 4(a). The input image is first divided into non-overlapping patches, each of which is treated as an individual token. These tokens are subsequently projected to a predefined feature dimension *C* through a linear embedding layer, enabling the subsequent transformer layers to more effectively capture relevant features.

In the proposed architecture, the input tokens are initially passed through a linear embedding layer and subsequently processed by a sequence of modified self-attention modules, known as swin transformer block. These modules operate within non-overlapping local windows to capture spatially localized features while maintaining the number of tokens. We define this initial structure – comprising the linear embedding and swin transformer block – as Stage 1, which serves as the basis for subsequent hierarchical processing.

To generate more efficient hierarchical representations, SwinT incorporates a patch merging mechanism [34]. As the network deepens, the number of tokens is progressively reduced through these patch merging layers, thereby improving the model’s computational efficiency. For instance, in Stage 2, features from each group of 2 × 2 neighboring patches are concatenated into a single vector of dimension 4*C*, followed by a linear layer that projects it down to 2*C* dimensions. This operation reduces the tokens count to one-fourth of the previous stage, effectively fusing local information for subsequent processing. The resulting representation, with a resolution of $\frac{H}{8}\times \frac{W}{8}$ , is then processed by additional swin transformer block.

This design is extended to Stage 3 and Stage 4, where the resolutions are further reduced to $\frac{H}{16}\times \frac{W}{16}$ and $\frac{H}{32}\times \frac{W}{32}$ , respectively, resulting in progressively abstract multi-dimensional representations. The complete formulation of the process is provided in Supplementary Information.

#### 2.2.2 Swin Transformer Block

SwinT enhances the model’s capacity for global information integration by incorporating a shifted window mechanism [34] into its architecture and constructing the swin transformer block, as illustrated in Figure 4(a). This module is a modification of the standard transformer block, in which the conventional multi-head self-attention (MSA) is replaced with localized attention mechanisms operating within regular and shifted windows – referred to as window-based MSA (W-MSA) and shifted window MSA (SW-MSA), respectively. This design facilitates cross-window information while significantly reducing computational complexity. The forward propagation of two consecutive swin transformer stacks is depicted in Figure 4(b) and the detailed algorithmic procedures are provided in Supplementary Information.

Specifically, each swin transformer block in the architecture comprises two primary components: a window-based self-attention module (either W-MSA or SW-MSA), and a two-layer multilayer perceptron (MLP) equipped with the GELU activation function. Layer normalization (LN) is applied before each submodule, while residual connections are employed following each submodule to enhance training stability in deep networks.

### 2.3 Training process

Data augmentation through geometric transformations of device patterns proves essential in preprocessing. A single simulation sample can generate 144 valid training samples via 4 flips, 4 rotations, and 3 translations, effectively expanding the original 20,000-sample training set to nearly 3 million samples while significantly reducing additional dataset generation costs. This operation substantially enhances prediction accuracy because neural networks struggle to inherently learn the rotational, reflectional, and translational symmetry properties of PCSEL photonic crystal layers from individual unit cells alone.

To validate this approach, we systematically evaluated POST model performance with different augmentation strategies (Table 3). The first two rows demonstrate improved *Q*-factor and SE prediction accuracy through rotation and flipping. While increasing translation iterations revealed oscillating accuracy patterns – with odd-numbered translations extracting more meaningful features – excessive translations may cause *R* ^2^ degradation due to premature overfitting. Notably, since training set size grows quadratically with translation iterations, we ultimately selected 4 flips, 4 rotations, and 3 translations to optimally balance training efficiency and model precision. It should be noted that “1 translation” here means no additional translation operation is performed.

**Table 3:** Data augmentation boosts accuracy.

Rotations & flips	Translation(s)	*R* ^2^ (SE)	*R* ^2^ (log *Q*)	Speed (s/epoch)
No	1	0.473	0.649	13
Yes	1	0.654	0.845	102
Yes	2	0.665	0.855	407
Yes	3	0.779	0.909	919
Yes	4	0.650	0.851	1,623
Yes	5	0.775	0.913	2,552
Yes	6	0.784	0.907	3,678

The table compares POST’s performance across different data augmentation strategies. The combination of 4 flips, 4 rotations, and 6 translations yields the best SE accuracy (*R* ^2^ = 0.784), while using 5 translations achieves the highest log *Q* accuracy (*R* ^2^ = 0.913). Translation = 1 means no additional translation operation is applied.

For the loss function, we employed the conventional mean squared error (MSE) (Equation (4)), while adopting the *R* ^2^ metric (Equation (5)) as our primary evaluation strategy to intuitively assess prediction accuracy and compare model performance between different photonic crystal properties. The *R* ^2^ metric provides an interpretable scale where: *R* ^2^ = 0 indicates that the model’s predictions are no better than simply using the mean of the property values, while *R* ^2^ = 1 represents perfect prediction accuracy. Higher *R* ^2^ values correspond to better predictive performance.

(4) $$\text{MSE}=\frac{1}{n}\overset{n}{\underset{i=1}{\sum }}{\left(y_{i}-{\hat{y}}_{i}\right)}^{2},$$

(5) $$R^{2}=1-\frac{\sum_{i=1}^{n}{\left(y_{i}-{\hat{y}}_{i}\right)}^{2}}{\sum_{i=1}^{n}{\left(y_{i}-\bar{y}\right)}^{2}}=1-\frac{\text{MSE}}{C^{*}},$$

where $C^{*}=\frac{1}{n}\sum_{i=1}^{n}{\left(y_{i}-\bar{y}\right)}^{2}$ is a dataset-dependent constant.

### 2.4 Model performance analysis

#### 2.4.1 Comparison to other neuron networks

A performance comparison of multiple existing neural networks is provided in Table 4 [26], [27], [35], [36], [37]. The evaluation includes not only traditional deep learning models (e.g., FCNN, CNN, and AlexNet) but also various ViT architectures, all tested on the same dataset. The table reveals that POST achieves the highest accuracy (highest *R* ^2^) in predicting both log *Q* and SE. For log *Q* prediction, POST attains an *R* ^2^ of 0.909, outperforming the second-tier models CaiT (0.883), ConViT (0.882), and LeViT (0.881). Similarly, in SE prediction, POST leads with an *R* ^2^ of 0.779, surpassing CaiT (0.763). These results indicate that POST’s architectural design excels at processing smaller-scale images and more accurately captures the physical features of photonic crystal lattice structures, whereas most ViT models exhibit significant advantages only when handling images larger than 100 pixels. Nevertheless, the second-tier ViT models still surpass the milestone model AlexNet in prediction accuracy, highlighting the overall superiority of ViT architectures.

**Table 4:** Comparison in accuracy and speed.

Neuron network	Train speed (s)	Predict speed (s)	*R* ^2^ of test set
**log *Q***			
FCNN	95	0.15	0.749
CNN	95	0.18	0.706
AlexNet [35]	492	0.51	0.817
DeiT-Ti [26]	433	0.52	0.869
CaiT-S24 [36]	1,630	1.69	0.883
ConViT-Ti [27]	743	0.92	0.882
LeViT-128s [37]	687	0.84	0.881
POST (this work)	918	1.08	0.909
**SE**			
FCNN	80	0.16	0.570
CNN	95	0.18	0.545
AlexNet [35]	492	0.51	0.667
DeiT-Ti [26]	432	0.52	0.749
CaiT-S24 [36]	1,621	1.70	0.763
ConViT-Ti [27]	743	0.91	0.731
LeViT-128s [37]	683	0.84	0.722
POST (this work)	920	1.09	0.779

The tables compare the prediction performance of different neural networks for two key PCSEL properties (log *Q* and SE), evaluating three critical metrics: training speed (seconds/epoch), prediction throughput (seconds/5 × 10^3^ samples), and test set *R* ^2^ scores.

It can be observed that traditional small-scale convolutional neural networks (CNNs) exhibit slightly lower predictive performance than fully connected neural networks (FCNNs). This is attributed to the fact that small CNNs, relying on convolutional mechanisms, can only identify local correlations between pixels and their neighbors, failing to extract global information. In contrast, analyzing the optical field of photonic crystals requires consideration of the entire unit cell structure.

While POST requires greater computational resources than lightweight models, its speed of >1 × 10^8^ samples/day and superior precision make it fully capable of supporting more optimization applications.

#### 2.4.2 Comparison between predictions and simulations

**Figure 5:**
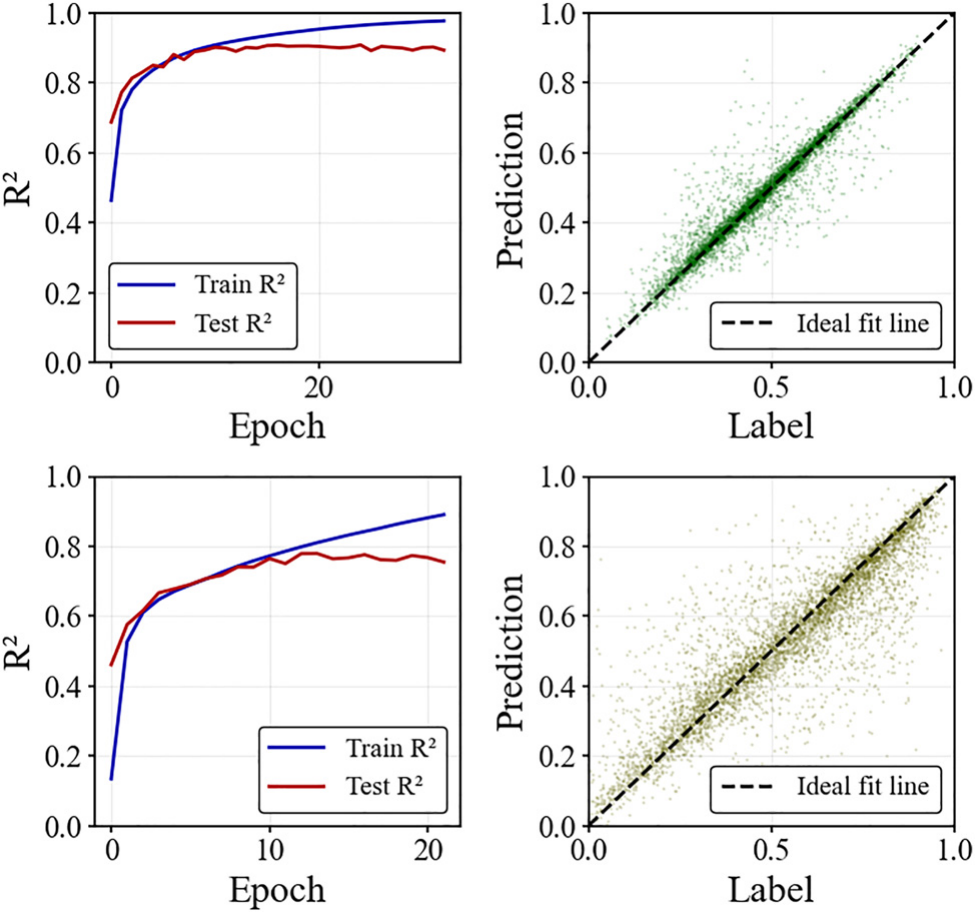
Training dynamics and predictive performance. Graphs (a) and (c) show the training curves of the POST’s predictions for log *Q* and SE, respectively. The blue line (Train *R* ^2^) and red line (Test *R* ^2^) indicate the goodness-of-fit of the model on the training set and test set as the training epochs progress. Graphs (b) and (d), respectively, display scatter plots of the model’s predictive performance for log *Q* and SE. The scatter points compare the model’s predicted values with the true values, while the black dashed line represents the ideal fit line used to evaluate prediction accuracy.

POST achieves prediction accuracies (*R* ^2^) of 0.909 for log *Q* and 0.779 for SE in PCSEL modeling (shown in Figure 5). It typically reaches peak accuracy within fifteen epochs, requiring only about 3 h of training time, followed by a slight overfitting trend that leads to a minor decline in test set performance.

The scatter plot visually confirms that POST’s prediction accuracy for log *Q* is significantly higher than for SE. This discrepancy stem from the more complex partial differential numerical solving process involved in SE calculations.

#### 2.4.3 Accuracy with limited training samples

**Figure 6:**
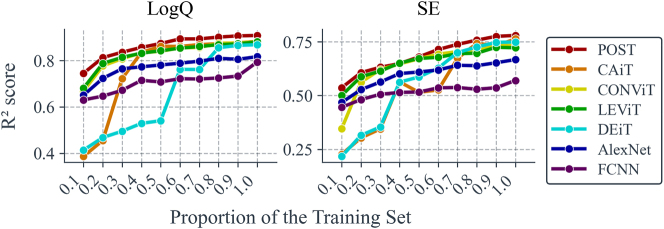
Accuracy versus training set size. The line chart compares the prediction accuracy (measured by *R* ^2^) of seven neural networks across varying training set sizes. The horizontal axis represents the proportion of the training set used, relative to the original size of 20,000 samples (80 % of the total 25,000 samples). The reduced training set is then augmented through 4 flips, 4 rotations, and 3 translations before training. The vertical axis shows the corresponding *R* ^2^ accuracy for each model.

For many simulation software tools, obtaining 25,000 raw data points remains challenging, especially when the input patterns for simulation modules have higher pixel density – simulation time costs increase quadratically. If the simulation input involves 3D structural data, the cost escalates cubically. Therefore, the learning performance of different neural networks under reduced raw data volumes (shown in Figure 6) is investigated, where the horizontal axis represents the proportion of the new training set relative to the original training set.

It can be observed that POST consistently maintains the strongest predictive capability across all dataset sizes and achieves *R* ^2^ accuracies of >0.8 for log *Q* and >0.6 for SE with only 20 % of the original training set. Additionally, we note that POST’s prediction accuracy for log *Q* converges with only about 60 % of the original training set, whereas its accuracy for SE may require a dataset larger than the original training set to converge. This also suggests that predicting the SE parameter is more complex than predicting *Q*.

Furthermore, training results with 20 % of the original training set in Figure 6 ( $R_{\mathrm{SE}}^{2}$ = 0.607 and $R_{\log Q}^{2}$ = 0.813) are worse than those without translation in Table 3 ( $R_{\mathrm{SE}}^{2}$ = 0.654 and $R_{\log Q}^{2}$ = 0.845). However, the former involved three additional translations in both horizontal and vertical directions, resulting in an actual training data volume that was significantly larger than the latter. This suggests that the augmented samples generated through translation carry less additional information compared to entirely new samples.

#### 2.4.4 Fourier-space feature attribution via SHAP analysis

To further investigate whether POST has internalized the physical priors embedded in CWT, we conduct a post hoc interpretability study using SHAP (SHapley additive explanations) [38], [39]. Unlike traditional saliency-based methods that rely on image-space gradients, this analysis evaluates the relative importance of Fourier components in predicting optical properties of PCSEL structures. Specifically, we apply a 2D discrete Fourier transform (DFT) to the photonic crystal unit cell and analyze the real and imaginary parts of selected Fourier coefficients as input features.

For a 32 × 32 dielectric constant distribution representing a photonic crystal pattern, we perform a 2D Fourier transform and extract a subset of its Hermitian-symmetric coefficients according to a triangular masking rule, as illustrated in Figure 2. This results in 512 unique complex-valued coefficients, each decomposed into real and imaginary parts and concatenated to form a 1,024-dimensional input vector. Each index (*m*, *n*) corresponds to a Fourier mode $\xi_{m,n}=\xi_{m,n}^{R}+i\xi_{m,n}^{I}$ .

To quantify the contribution of each Fourier component to the model’s predictions, we apply the Permutation SHAP algorithm with 50 background samples and 100 evaluation samples drawn from the test dataset. The prediction function internally recovers the spatial-domain input from each perturbed Fourier vector, enabling seamless compatibility with the original POST architecture trained in the spatial domain.

**Figure 7:**
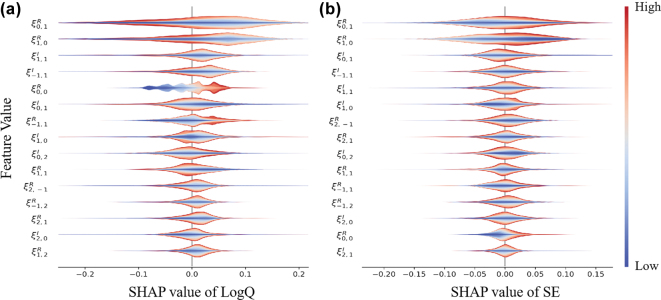
Distributions of SHAP values. Distributions of SHAP values for the top 15 Fourier coefficients contributing to the prediction of (a) SE and (b) log *Q*. Each row denotes a distinct coefficient in the Fourier domain, with the *x*-axis showing its corresponding SHAP value. Color shading indicates the relative magnitude of the coefficient, with red and blue representing high and low values, respectively. The violin plot contours represent the variability and concentration of each coefficient’s contribution. Positive SHAP values suggest a positive influence on the predicted outcome, while negative values imply a suppressive effect.

Figure 7 shows the SHAP violin plots for the top 15 most influential Fourier features for both SE and log *Q*. The most impactful coefficients are concentrated near the center of the Fourier domain, reflecting their critical roles in determining photonic crystal performance.

In particular, $\xi_{0,0}^{R}$ corresponds to the average refractive index of the photonic crystal layer. A higher value typically indicates a lower filling factor, meaning more high-index material is present. This leads to weaker photonic crystal modulation but stronger waveguiding effect, which enhances vertical optical confinement and contributes positively to *Q*.

Coefficients such as $\xi_{1,0}^{R}$ and $\xi_{0,1}^{R}$ are directly related to vertical radiation coupling. Deviations of their values from zero increase surface emission efficiency (SE) by enhancing out-of-plane leakage. However, since this also introduces greater radiation loss, it tends to reduce *Q*.

In contrast, $\xi_{-1,1}^{R}$ governs the strength of in-plane two-dimensional diffraction, contributing to lateral distributed feedback. A larger magnitude of this coefficient suggests stronger horizontal coupling, which reinforces resonant feedback and increases *Q*.

Although the top-ranked Fourier features differ slightly between SE and log *Q*, substantial overlap exists in high-impact modes. This indicates that both performance metrics are shaped by a common set of structural features, especially those affecting radiative loss, confinement, and feedback within the photonic crystal.

According to the 3D-CWT framework [40], the radiation constant *α* and thus the *Q*-factor of the lasing mode depend explicitly on three quantities: the non-Hermitian coupling coefficient *μ*, and the real and imaginary parts (*R*, *I*) of the effective Hermitian coupling $\left(\kappa_{1D}+\kappa_{2D-}\right){\text{e}}^{-\text{i}\theta_{\text{pc}}}$ . Specifically,

(6) $$Q\approx \frac{2\pi /a}{\alpha },\;\alpha =\frac{\mu }{\mu^{2}+R^{2}}I^{2},$$

where *a* is the lattice constant. The coupling coefficients *κ*_1*D*_ and *κ*_2*D*−_ are positive correlated with the Fourier coefficients *ξ*_1,−1_ (or *ξ*_−1,1_) and *ξ*_2,0_ (or *ξ*_0,2_), respectively. Therefore, the *Q*-factor is highly sensitive to the magnitudes of *ξ*_−1,1_ and *ξ*_2,0_. This theoretical insight aligns well with the SHAP analysis, which identifies $\xi_{-1,1}^{R}$ can significantly influence *Q* predictions.

It should be noted that the ultimate performance of a PCSEL is governed by the combined action of Hermitian couplings and non-Hermitian coupling [40]. In this context, our SHAP analysis does not aim to replace the underlying physical derivations, but rather to evaluate the interpretability of POST. By highlighting that the most influential Fourier components align with those known to control (*R*, *I*, *μ*) in coupled-wave theory, SHAP provides evidence that POST has internalized meaningful physical priors, thereby enhancing trust in the model’s predictions.

### 2.5 Conclusions

The authors employed a novel neural network, POST, to predict the photonic crystal simulation results of the CWT model. This approach enables fully automated and highly accurate predictions across single, double, and even triple lattices, as well as various non-circular complex hole structures. It can complete nearly 10,000 predictions in just 2 s, with a mean squared error less than 50 % of previous similar work. Moreover, using only 20 % of the original dataset, it achieves prediction accuracy surpassing prior studies. And SHAP analysis confirms that the model prioritizes physically meaningful Fourier components indicating alignment with CWT theory.

These results demonstrate that POST not only accelerates PCSEL evaluation but also captures key physical principles, making it a promising tool for future AI-assisted photonic device design. The dataset used will be released to support broader research efforts.

Looking forward, an important extension of this work is to validate and retrain POST on more comprehensive data sources beyond CWT. Because POST’s vision-transformer architecture is data-driven and not tied to a specific physics model, it can in principle be re-trained or fine-tuned using full-wave simulation data (e.g., FDTD or FEM (Finite element method) results such as threshold mode-spacing and far-field beam quality) or even experimental measurements. This architectural flexibility means that POST could serve as a high-speed surrogate for diverse modeling approaches, bridging the gap between analytical approximations and real-world device behavior.

## 3 Methods

### 3.1 CWT-based dataset generation

The optical properties of PCSELs were simulated using a custom three-dimensional coupled-wave theory (3D-CWT) solver implemented in Python. The model accounts for both in-plane diffraction and vertical radiation loss, using up to 441 Fourier harmonics to ensure numerical convergence. For each PCSEL configuration, SE and log *Q* were computed. The refractive indices used in the simulations are listed in Table 1. The photonic crystal cell was discretized on a 32 × 32 grid, and simulations were repeated for over 25,000 unique photonic crystal geometries. The simulated PCSEL devices consist of a finite-size photonic crystal pattern of 200 × 200 μm. The finite-size photonic crystal is discretized to a 17 × 17 grid for CWT calculation, where the underlying partial differential equations are mathematically solved by the FEM method.

### 3.2 Neural network model: POST

The POST model is based on the SwinT architecture and was implemented using PyTorch. The model takes a single-channel 32 × 32 real-space dielectric pattern as input and passes it through four hierarchical self-attention stages. For training, we used an Adam optimizer with a learning rate of 10^−4^ and batch size of 64. Separate models were trained for SE and log *Q* using mean squared error loss. Training and evaluation were performed on a single NVIDIA RTX 3070 GPU.

Although POST is trained using real-space dielectric patterns, an alternative approach is to directly use Fourier coefficients as model input. Fourier-space features naturally encode Bragg scattering and mode coupling information, providing a physically transparent representation. In preliminary tests, we found such Fourier-based inputs to be effective in reproducing device behavior [32]. Nevertheless, we intentionally selected real-space images for the present work. This vision-based representation enables POST to scale to arbitrary geometries or full-wave simulation and to exploit state-of-the-art computer vision architectures such as the swin transformer, which are optimized for spatial correlation learning. As a result, real-space inputs offer greater flexibility and generalizability for practical PCSEL design automation, while Fourier-space features remain a promising complementary representation for future extensions.

### 3.3 SHAP analysis in the Fourier domain

To interpret the model’s prediction behavior, we used the PermutationExplainer from the SHAP Python library [39]. SHAP values were computed in the Fourier domain using 50 background samples and 100 test samples. The model’s internal prediction logic includes inverse Fourier recovery to spatial domain before inference. Violin plots of SHAP values (Figure 7) were used to identify the most influential modes for SE and log *Q*.

## Supplementary Material

Supplementary Material Details
