# Supplementary material for: POST: photonic swin transformer for automated and efficient prediction of PCSEL
Source: Nanophotonics. 2025 Sep 30;14(22):3599–610. doi: 10.1515/nanoph-2025-0317 (PMC12592633; doi:10.1515/nanoph-2025-0317)
Supplement: Supplementary file 1 — Supplementary Material Details [file j_nanoph-2025-0317_suppl_001.pdf]

Supplementary Information of POST: Photonic Swin Transformer for Automated and Efficient Prediction of PCSEL

Qi Xin[1,2†], Hai Huang[1,2†], Chenyu Li[1], Kewei Shi[3], Zhaoyu Zhang[1,2*]

[1]School of Science and Engineering, The Chinese University of Hong Kong, Shenzhen, Guangdong 518172, China.
[2]Guangdong Key Laboratory of Optoelectronic Materials and Chips and Shenzhen Key Lab of Semiconductor Lasers, School of Science and Engineering, The Chinese University of Hong Kong, Shenzhen, Guangdong 518172, China.
[3]The University of Hong Kong, Pok Fu Lam Road, Hong Kong, China.

*Corresponding author(s). E-mail(s): zhangzy@cuhk.edu.cn;
Contributing authors: qixin@link.cuhk.edu.cn; haihuang@link.cuhk.edu.cn; chenyuli@link.cuhk.edu.cn; kewei.shi@connect.hku.hk;
†These authors contributed equally to this work.

1 Algorithm of Swin Transformer

Algorithm 1 Architecture of the Encoder Based on Swin Transformer Blocks

Input: Image $I \in \mathbb{R}^{H \times W \times 1}$, depths $\{d_1, d_2, d_3, d_4\}$, predefined feature dimension C
$X \leftarrow \text{PatchPartition}(I)$
$\mathcal{Z}_0 \leftarrow \text{LinearEmbed}(X)$ ▷ $\mathcal{Z}_0 \in \mathbb{R}^{\frac{H}{4} \times \frac{W}{4} \times C}$
$\mathcal{Z}_1 \leftarrow \text{SwinBlock}_1^{(d_1)}(\mathcal{Z}_0)$
for $i = 2$ to 4 **do**
  $\tilde{\mathcal{Z}}_{i-1} \leftarrow \text{PatchPartition}(\mathcal{Z}_{i-1})$ ▷ $\tilde{\mathcal{Z}}_{i-1} \in \mathbb{R}^{\frac{H}{2^{i+1}} \times \frac{W}{2^{i+1}} \times 2^i C}$
  $\mathcal{Z}_i \leftarrow \text{SwinBlock}_i^{(d_i)}(\tilde{\mathcal{Z}}_{i-1})$
end for
return $\mathcal{Z}_4$

Algorithm 2 Forward Propagation of Consecutive Swin Transformer Blocks

Input: Feature z^{l-1} at stage $l-1$, shift size S, attention heads h
Stage l: Window-based Multi-Head Self-Attention (W-MSA)
$x_1^l \leftarrow \text{LN}(z^{l-1})$
$Q, K, V \leftarrow x_1^l W^q, \quad x_1^l W^k, \quad x_1^l W^v$
for $i = 1$ to h **do**
  $\text{head}_i \leftarrow \text{softmax}\left(\frac{Q_i K_i^T}{\sqrt{d_k}} + B\right) V_i$
end for
$\text{W-MSA} \leftarrow \text{concat}(\text{head}_1, \ldots, \text{head}_h) W^o$
$\hat{z}^l \leftarrow \text{W-MSA} + z^{l-1}$
$x_2^l \leftarrow \text{LN}(\hat{z}^l)$
$z^l \leftarrow \text{MLP}(x_2^l) + \hat{z}^l$

Stage $l+1$: Shifted Window Multi-Head Self-Attention (SW-MSA)
$x_1^{l+1} \leftarrow \text{LN}(z^l)$
$x_{1,\text{shifted}}^{l+1} \leftarrow \text{shift}(x_1^{l+1}, S)$
$Q', K', V' \leftarrow x_{1,\text{shifted}}^{l+1} W^q, \quad x_{1,\text{shifted}}^{l+1} W^k, \quad x_{1,\text{shifted}}^{l+1} W^v$
for $i = 1$ to h **do**
  $\text{head}'_i \leftarrow \text{softmax}\left(\frac{Q'_i (K'_i)^T}{\sqrt{d_k}} + B + M\right) V'_i$
end for
$\text{SW-MSA} \leftarrow \text{concat}(\text{head}'_1, \ldots, \text{head}'_h) W^o$
$\hat{z}^{l+1} \leftarrow \text{SW-MSA} + z^l$
$x_2^{l+1} \leftarrow \text{LN}(\hat{z}^{l+1})$
$z^{l+1} \leftarrow \text{MLP}(x_2^{l+1}) + \hat{z}^{l+1}$

In **Algorithm 1**, PatchPartition($\cdot$) denotes the patch merging operation, and $\text{SwinBlock}_i^{(d_i)}$ represents the i-th Swin Transformer module, where the depth parameter d_i determines the number of Swin Transformer blocks stacked within each stage.

In our implementation, to avoid overfitting and computational redundancy caused by excessive stacking, while maintaining sufficient model capacity, we adopt a configuration in which each stage contains two Swin Transformer blocks, i.e., $(d_1, d_2, d_3, d_4) = (2, 2, 2, 2)$. In addition, the value of predefined feature dimension C is set to 96. This setting demonstrates favorable fitting efficiency and stability across multiple experiments.

In **Algorithm 2**, The numbers of attention heads for the four successive stages are set to $(3, 6, 12, 24)$, allowing the model to capture features at different levels of abstraction. To balance accuracy and computational cost, we set the window size H to 7 and adopt a shift size $S = 2$ to enhance the model's ability to capture local interactions at window boundaries.

An attention mask M is introduced to constrain computations within individual windows[1], while the relative position bias matrix B encodes spatial relationships between tokens. These components jointly enhance the model's ability to incorporate positional information, improving the reliability of the final predictions.

References

[1] Liu, Z., Lin, Y., Cao, Y., Hu, H., Wei, Y., Zhang, Z., Lin, S., Guo, B.: Swin transformer: Hierarchical vision transformer using shifted windows. In: Proceedings of the IEEE/CVF International Conference on Computer Vision, pp. 10012–10022 (2021)
